# The rK39 strip test is non-predictor of clinical status for kala-azar

**DOI:** 10.1186/1756-0500-2-187

**Published:** 2009-09-22

**Authors:** Dharmendra P Singh, Shyam Sundar, Tribhuban M Mohapatra

**Affiliations:** 1 Institute of Medical Sciences, Banaras Hindu University, Varanasi, UP, India; 2 Department of Medicine, Institute of Medical Sciences, Banaras Hindu University, Varanasi, UP, India; 3 Department of Microbiology, Institute of Medical Sciences, Banaras Hindu University, Varanasi, UP, India

## Abstract

**Background:**

The rK39 strip test is reported to be simple, sensitive, specific, non-invasive and economical test. Since this method is supposed to be patient friendly, it may easily be accepted for sero-epidemiological surveys. An attempt was made to evaluate the role of rK39 strip test in pre and post treatment phases of Kala azar, as a diagnostic and prognostic marker, in addition to other laboratory investigations, in order to evaluate its role in sero-epidemiological surveys.

**Findings:**

A total of 210 cases were selected for the study. One hundred clinically and parasitologically confirmed cases were corroborated with other hematological profiles. The formol-gel test was included along with well matched control group comprising of normal endemic controls (50), non-endemic normals (20) and other febrile cases (40). All groups were tested by rK39 strip test. Fifty Kala azar cases were followed up after completion of successful treatment. They were subjected to rK39 strip test after 0, 90 and 180 days of completion of successful treatment.

The rK39 showed sensitivity, specificity, PPV, NPV, and diagnostic accuracy of 98% (95% CI 91.7-100), 100%, 100%, 90% (95% CI 66-100) and 98% (95% CI 92.6-100) respectively. All the 50 cured followed up cases showed positive result by rK39 strip test even after 180 days of completion of successful treatment.

**Conclusion:**

The test seems an ideal qualitative test for the diagnosis of kala-azar. But for sero-epidemiological studies the test may be used with other parameters. Alternatively a quantitative ELISA using rK39 antigen may be used.

## Background

Visceral Leishmaniasis (VL) is a potentially fatal disease, caused by *Leishmania donovani *complex. The disease is endemic in 88 countries spread over 5 continents with a total of 12 million cases and 350 million people at risk [1]. Of all the VL cases, more than 90% are from India, Nepal, Bangladesh, Southern Sudan and Northeast Brazil. In India, VL is endemic in Bihar, Eastern part of India, Tamil Nadu, Jamu and Kashmir.

For decades, detection of the parasite has been considered to be the definitive diagnosis of this disease. It requires invasive procedure to detect the parasite in the tissue, i.e. spleen, bone-marrow and lymph node. Although it is the gold standard for the diagnosis of VL but the procedure is cumbersome, risky and very difficult to apply in field conditions. To obviate these procedures various serological tests have been developed, evaluated and tried. These tests have the advantage of being safe, less invasive and can be done in large numbers of samples. Nonspecifc methods, such as Napier's formol gel (aldehyde test) and the Chopra antimony test, which depend upon raised globulin levels, have been used in the diagnosis of VL for several decades. Lack of specificity, as well as varying sensitivities, render them highly unreliable. In the past, conventional methods of antibody detection like immunodiffusion [2], complement fixation test [3], indirect hemagglutination test and countercurrent immunoelectrophoresis [4] were being used as diagnostic tool for VL. However, apart from practical difficulties at peripheral laboratories, the sensitivities and specificities of most of the above tests had been the limiting factors. These tests are no longer in use for routine diagnosis of VL. Indirect fluorescent antibody test was found to be highly sensitive and specific but difficult to apply in field conditions. ELISA is a versatile test. It is easy to apply and highly sensitive but its specificity depends on the type of antigen used [5]. Latex agglutination test (KATEX), for the detection of leishmanial antigen in urine of patients with VL showed high specificity but equivocal sensitivity [6]. Direct agglutination test (DAT) is a sensitive test and well adapted to screening of a large population, especially using its variant referred to as, "Fast Agglutination Screening Test". It has been advocated for field use [7-9]. However, simple format of rK39 strip test suggests its clear advantages for use in peripheral health services compared to the current FD-DAT format. The rk39 strip test is a simple, rapid and accurate test with good sensitivity and specificity, which can be used without any specific expertise. Recently, some authors have advocated its use for the sero-epidemiological surveys [10].

## Methods

The study was carried out in Muzaffarpur district of Bihar state, India, for comparative evaluation of the sensitivity, specificity, predictive values (positive and negative) and diagnostic accuracy of rK39 test. A total of 210 subjects were investigated. The study group comprised of 100 clinically, hematologically and parasitologically confirmed patients, 50 endemic normals (mostly apparently healthy first degree relative of patients residing in the endemic area), 20 healthy individuals from Europe where kala-azar is not endemic (non endemic controls) and a group of 40 individuals with history of febrile illness (tuberculosis, malaria and enteric fever) were investigated for kala-azar. All individuals were subjected to rK39 strip test. The cases presented had clinical symptoms and signs suggestive of kala-azar i.e. fever, splenomegaly and anaemia. Routine investigations were carried out to exclude other etiologies of the fever with hepato-splenomegaly. The final diagnosis of VL was established by Karnofsky score, WBC count, hemoglobin assay, fomol-gel test and demonstration of parasites in splenic aspirates. These patients were bled on first encounter before and after specific treatment. All patients were provided a standard treatment with amphotericin B (Sarabhai Chemicals, Vadodara, India) in 1 mg/kg infusions on alternate days up to a total of 15 infusions.

After completion of treatment the patients were free of symptoms. The leucocytes count restored and the formol-gel test showed a tendency of negative reaction. Out of 100 treated Kala azar cases, 50 cases (easily accessible) were further followed up after elaborate scrutiny, to asses the rK39 antibodies by strip test. Approximately 5 to 6 ml of blood was collected from each treated patient and subjected to rK39 strip test for demonstration of antibodies. Easily accessible among treated patients were monitored every 3 months for 6 months by rK39 strip test (0, 90, & 180 days). Their case history were documented.

Formol-gel test was carried out by adding a drop of formalin (40%) in two ml of serum and incubated for 20 minutes; a positive result was indicated by appearance of jellification of milky white opacity.

The rK39 strip was provided by In Bios International, Inc. 562 1^st ^Ave. South, suite 600 Seattle, WA 98104 USA. The strip for VL assay is a qualitative membrane based immunoassay for the detection of antibodies to visceral leishmaniasis in human serum. The test was conducted according to instruction provided by manufacturers. In brief, 20 μl serum was placed on pad. Following this, 2-3 drops of wash buffer (provided) was added to pad and the mixture was allowed to migrate up the strip by capillary action. The result was read within 10 minutes. There are two pink lines in a positive result; the upper one is control and lower one is the test. The appearance of only one pink line means a negative result when this is upper line; but if the one pink line is the lower line (test) this means an invalid result.

### Statistical analysis

The serological data thus obtained were statistically analyzed after entry in EPI-info (version-6) and SPSS statistical package. The Fleiss Quadratic method was used for the calculation of confidence interval of proportions. The sensitivity, specificity, predictive values (positive and negative) and diagnostic accuracy were mathematically calculated as follows: sensitivity = (true positive/true positive + false negative) × 100; Specificity = (true negative/true negative + false positive) × 100; Positive predictive value (PPV) = (true positive/total positive) × 100; Negative predictive value (NPV) = (true negative/total negative) × 100; Diagnostic accuracy = (true positive + true negative/grand total) × 100.

The study was approved by the Ethical committee of Institute of Medical Sciences, Banaras Hindu University. Written consent was obtained from all the subjects.

## Results

Of the 100 parasitologically proven index cases, 98 cases were found to be positive for rK39 antibodies. Among the apparently healthy endemic normals10% (5 out of 50) showed positivity for rK39 strip test while none from non-endemic area showed positive reaction. No cross reactivity was found between VL cases and other febrile cases. A detailed clinical and laboratory findings along with the rK39 results has been depicted in the Table 1. It was observed that the rK39 strip test had sensitivity, specificity, PPV, NPV, and diagnostic accuracy of 98% (95% CI 91.7-100), 100%, 100%, 90% (95% CI 66-100) and 98% (95% CI 92.6-100) respectively.

**Table 1:** Clinical and laboratory features along with rK39 results of patients with visceral Leishmaniasis and Controls

**Study entry**			
	**VL patients**		**Controls**
	**Pre-treated**	**Post-treated (180 day)**	
No.	100	50	110
Age (years)	26 ± 4.5	-	27 ± 4.1
Duration of illness (months)	4.4 ± 0.45	-	-
Weight (kg)	40.1 ± 6.6	45.4 ± 5.2	60 ± 5.4
Karnofsky score	71.8 ± 2.5	85.4 ± 2.7	100
Spleen size(cm)	8.4 ± 2.4	4.0 ± 0.6	-
Splenic aspirate score	2.9 ± 0.4	-	-
WBC count(× 10^3^/mm^3^)	3.49 ± 0.59	6.4 ± 0.6	9.1 ± 2.3
Hemoglobin (g/dL)	6.9 ± 0.48	11.2 ± 0.76	14.2 ± 2.1
Platelet count(× 10^3^/mm^3^)	72 ± 0.35	270 ± 0.5	254 ± 23
rK39 positivity	98*	50*	5*

*indicates total no. of positive.

**Figure 1:**
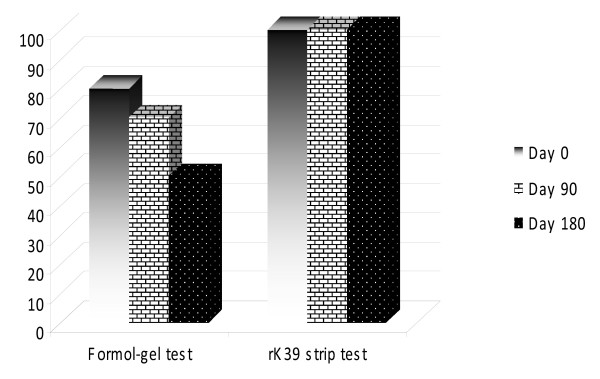
**Comparative evaluation of Formol-gel test and rK39 strip test in post treated cases**.

The rK39 strip test showed positive result in all the 50 cases after 180 days whereas formol-gel test showed 80%, 70%, and 50% positive result after 0, 90, 180 days respectively (Figure 1).

## Discussion

The present study was conducted on 210 subjects to evaluate rK39 strip test as a diagnostic and prognostic tool for kala-azar. It showed 98% sensitivity and 100% specificity with confirmed VL cases whereas it showed negative reaction with non-endemic normals. Five subjects from an endemic area were found to be positive by rK39 strip test. This may be due to the fact that these persons belonged to the same area having the same environment. The presence of antibody in these cases may indicate either subclinical infection or they may be during the incubation period of the disease or they were partially treated by the local quacks [11]. On the contrary, all the febrile cases showed non-reactive results by this method. This result corroborates with the findings of other workers, thereby suggesting that it is an ideal format for use under field conditions as it is quick and no special equipment is needed [12,13]. On the other hand, the study conducted in Sudan showed lower sensitivity and specificity of this test [14]. The reason for this difference in sensitivity may be due to difference in antibody responses observed in different ethnic groups [10,15]. With 98% diagnostic accuracy and 100% PPV as observed in this study, it proves that rK39 strip test is a good diagnostic tool qualitatively.

In the present study, fifty successfully treated individuals were followed up for six months. All these patients showed positive reaction by rK39 strip till 6 months. It indicates persistence of antibodies against rK39 antigen. This observation was supported in some studies performed in successfully treated cases for the detection of antibodies against rK39 antigen by ELISA [13,16].

In its present form, the rK39 strip test has the potential to be used as a suitable method for diagnosis of VL. For screening as a sero-epidemiological tool it has some important limitations as antibody detection cannot discriminate between past and current infection. Positive result may be due to previous infection, relapse or subclinical infection. It may not have relevance in predicting the current status of the disease in an epidemiological survey. Since some authors [10] advocated the use of this test in epidemiological surveys it may not be addressed alone. Rather, the test may be corroborated with appropriate clinical examination and patient interview.

## Conclusion

This test used as such can only give an estimated idea about the proportion of people that have had exposure to leishmania parasite which eventually may suggest the transmission dynamics of the disease that can be used for working out the disease control strategies. An alternative method such as semi quantitative rK39 strip test or quantitative ELISA with rK39 antigen may be useful for sero-epidemiological surveys.

## Competing interests

The authors declare that they have no competing interests.

## Authors' contributions

DPS conducted the study as part of thesis for the degree of master of Medicine and provided expertise of data analysis and manuscript preparation. SS provided the clinical cases and data. TMM supervised the study and provided expertise in statistical analysis and manuscript preparation. All authors read and approved the revised final manuscript.
